# Development of an Essential Information Set for Supporting Life With Bipolar Disorder: A Modified Delphi Study With Patients, Families and Healthcare Professionals

**DOI:** 10.1111/hex.70470

**Published:** 2025-10-22

**Authors:** Rieko Nagata, Takashi Amagasa, Takashi Okura, Kayoko Ichikawa, Yoshitaka Nishikawa, Mayumi Toyama, Hiroshi Okada, Yoshimitsu Takahashi, Yu Sakagami, Eiji Suzuki, Norio Ozaki, Takeo Nakayama

**Affiliations:** ^1^ Department of Health Informatics, School of Public Health Kyoto University Graduate School of Medicine Kyoto Japan; ^2^ Shizuoka Graduate University of Public Health Shizuoka Japan; ^3^ Department of Health and Welfare, Graduate School of Health and Welfare Science Okayama Prefectural University Soja Japan; ^4^ Department of Preventive Medicine Kyoto University Graduate School of Medicine Kyoto Japan; ^5^ Occupational Welfare Division, Agency for Health, Safety and Environment Kyoto University Kyoto Japan; ^6^ Department of Psychiatry Tohoku Medical and Pharmaceutical University Sendai Japan; ^7^ Pathophysiology of Mental Disorders Nagoya University Graduate School of Medicine Nagoya Japan

**Keywords:** attitudes, bipolar disorder, consensus development, Delphi technique, health knowledge, patient participation, practice, psychoeducation

## Abstract

**Objective:**

Bipolar disorder is a chronic condition characterised by alternating periods of depression and heightened mood, leading to considerable disability and frequent relapses, often resulting in difficulties in maintaining a stable social life. This study aimed to develop a set of essential information through a structured consensus process with patients, family members and healthcare professionals, to support patients with bipolar disorder to actively participate in their treatment and maintain a social life.

**Methods:**

A modified Delphi method was used to develop a consensus among patients, family members and healthcare professionals. The following steps were involved: a review of existing relevant literature, individual rating by experts, and multiple expert panel meetings. We conducted two rounds of individual ratings and face‐to‐face panel meetings following the literature review.

**Results:**

The literature review identified 126 candidate items. The expert panel comprised 11 members, including patients, family members and healthcare professionals. Through the consensus process, 23 items were selected to form the essential information set.

**Conclusion:**

The developed 23‐item essential information set provides the minimum necessary information for patients, family members and healthcare professionals to support living well with bipolar disorder. This set can serve as a basis for psychoeducation. This study provides a foundation for future research to explore its effectiveness in clinical settings.

**Patient or Public Contribution:**

Patients and family members participated in the expert panel, where they rated candidate items, shared their lived experiences and provided feedback on the wording of each item in the information set during the panel meetings.

## 1 Background

Bipolar disorder is a chronic condition with recurrent episodes of mania and depression, affecting approximately 1% of the global population and 0.6% in Japan [1, 2]. Manic symptoms can severely impact social and occupational functioning, and lack of accessible and available care and stigma can contribute to poorer health and social outcomes, while depressive symptoms can result in enduring social challenges even after symptoms resolve, such as a hindrance to long‐term employment [3]. With the 5‐year recurrence rate reported to be as high as 81%–91% [4], bipolar disorder is associated with one of the highest suicide rates among psychiatric disorders [5]. Moreover, an association between the number of bipolar episodes and an increased risk of dementia has been noted [6]. Thus, the long‐term goal in treating bipolar disorder is to improve treatment adherence and prevent the recurrence of both manic and depressive episodes [7].

Combining psychosocial interventions (cognitive‐behavioural therapy, family therapy, interpersonal and social rhythm therapy, etc.) with medication can effectively reduce the recurrence rate [8]. In addition, both domestic and international guidelines recommend psychoeducation, which involves acquiring knowledge related to the illness, continuing medication therapy and creating an environment that makes symptom recurrence less likely through self‐management. Despite the existence of effective psychoeducation programmes for mental illness and bipolar disorder, such as the Psychoeducation Manual for Bipolar Disorder [9], programmes are rarely accessible in routine clinical practice in Japan due to the need for specialised knowledge and human resources.　According to a report by Ono [10], a survey of medical institutions with psychiatric departments in Japan revealed that only 6% of the facilities reported being adequately equipped to provide psychotherapy. The Japanese Practice Guidelines for Bipolar Disorder [7] pointed out this gap and suggested psychoeducation focused on ‘the minimum essential information’ during the maintenance treatment initiation phase (or early after improvement of acute symptoms) to address the resource shortage and suggested the seven information categories: (1) Maintain a regular lifestyle. (2) Identification of factors leading to worsening of the disease condition. (3) Management of issues having an adverse impact. (4) Identification of signs of new relapse and formulation and implementation of preventive measures. (5) Eliminate misconceptions and stigma about the disease. (6) Achieve effective drug therapy. (7) Dealing with substance abuse and anxiety. However, the guideline does not mention the details of the information that should be provided to patients and family members. Therefore, there is a significant gap in the availability of practical psychoeducational resources that can be easily implemented in clinical settings.

For individuals living with mental disorders, being informed about their illnesses and treatments has been shown to be beneficial, leading to better treatment adherence, early symptom detection, enhanced self‐management, decreased self‐stigma and increased acceptance of the illness [11, 12, 13, 14]. Moreover, shared decision‐making has been recognised to be crucial in selecting treatment choices to improve treatment adherence [15], which is also endorsed by the Japanese clinical practice guideline [7]. Indeed, obtaining basic information about the disease and its treatment is important for patients to actively participate in their treatment plans.

While patients obtain information about disease and treatment primarily from healthcare professionals, research indicates that only about one‐third of patients find such information given by healthcare professionals helpful [16]. One reason for this is the insufficient communication between patients and their physicians; physicians often provide a large amount of information in a limited time, making it difficult for patients to digest the information at their own pace [14]. Although in some cases, pamphlets for patients and their families are made available by healthcare professionals or pharmaceutical companies to facilitate the understanding of disease and treatment, prioritising information from the patient's view has not been well discussed. Patients and their families often turn to sources other than healthcare professionals, with Internet searches and social media being a common choice. However, amidst a flood of mixed information from healthcare professionals and personal accounts from patients and families, it can be challenging to obtain scientifically accurate and individualised information [16, 17].

A previous study found that difficulty in assessing patients' understanding was another reason why information from healthcare professionals was often not seen as helpful [16]. Stump et al. developed the Bipolar Disorder Knowledge Scale (BDKS) that comprised 25 items with six domains (risk/cause of onset, diagnosis, disease course, symptoms, treatment and the life impact of the disease) [18] to measure the level of knowledge about bipolar disorder in a general population. Although the BDKS is meaningful in aiming to construct a common ground to understand bipolar disorders, it emphasises scientific knowledge of the disease. Patients and families also need information on how to reduce the risk of relapse and how to live with bipolar disorder. Given these challenges, engaging a diverse group of stakeholders, including patients and carers, in research processes may offer valuable insights. In the context of bipolar disorder, Majid et al. reported a successful development of self‐monitoring technology through the active participation of patients [19].

The aim of this study is to address this gap by collaboratively developing an essential information set (hereinafter, the ‘essential information set’) to enhance communication between healthcare professionals and patients, thereby supporting patients to live well with the disease. We hypothesised that the proposed set mitigates the issue of limited consultation time by providing structured and prioritised information that can be easily communicated during clinical encounters. This approach ensures that patients receive the most critical information without being overwhelmed, thereby improving their understanding and engagement in their treatment.

## 2 Methods

### 2.1 Overview of Development of Essential Information Set

**Figure 1:**
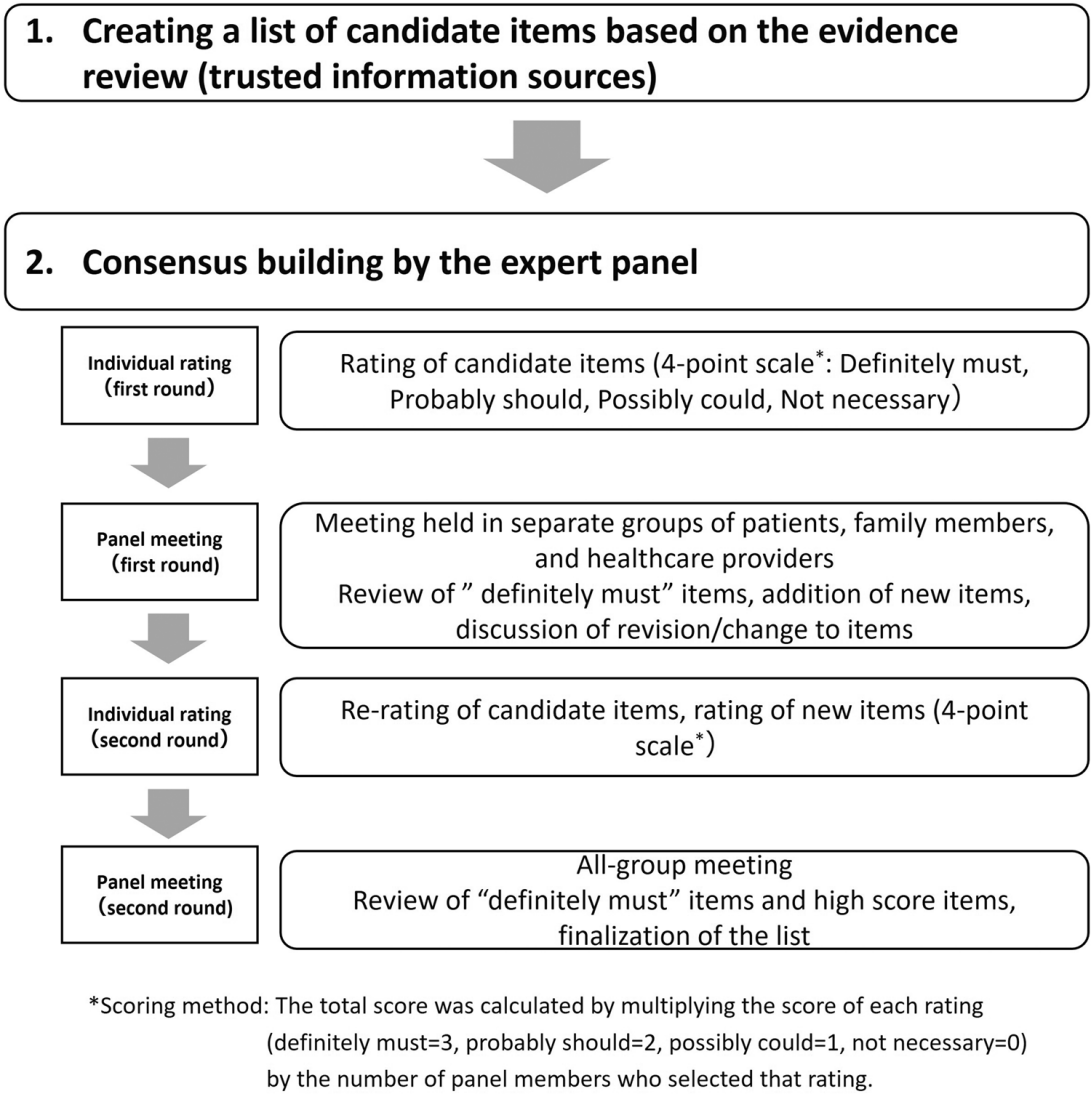
Overview of methodology.

We used a modified Delphi method to develop a consensus among patients, family members and healthcare professionals. This method, widely used in developing practice guidelines and quality indicators [20, 21], consists of a review of existing relevant literature, individual rating by experts, and multiple expert panel meetings. To incorporate diverse perspectives, we conducted two rounds of individual ratings, followed by face‐to‐face panel meetings, according to previous studies that adopted the modified Delphi method [22]. Patients and families are generally reluctant or hesitant to speak out against healthcare professionals, whose views on the disease may differ from those of patients and their families. For this reason, we decided to hold the first panel meeting separately for each group (i.e., patients, family members and healthcare professionals), while the second panel meeting was conducted with all members present to reach a final consensus (Figure 1).

This study was approved by the Kyoto University Graduate School of Medicine and Faculty of Medicine Medical Ethics Committee (R3310‐1) and conforms with the Declaration of Helsinki. Informed consent was obtained from all study participants. The consent form included a statement explaining that participants might experience discomfort due to the nature of the items being evaluated or the discussions during the panel meeting. It also recommended that participants confirm with their physicians that they were suitable to take part in the study. Moreover, participants were offered contact information in case any issues arose. Advice was obtained from psychiatrists (E.S., N.O. and T.K.) who are directors of the Japanese Society of Mood Disorders during each step of the consensus‐building process.

### 2.2 Generation of Initial Set of Essential Information

The following criteria were used to select candidate items to generate an initial set of information for individual ratings.

– Information from academic societies or government agencies in Japan or other countries, or pharmaceutical companies that commercialise drugs for bipolar disorder in Japan
– Information for patients or family members living with bipolar disorder
– Information available on the Internet

In addition to the above, we referred to the content of a psychoeducation programme [23] recommended in the 2020 Japanese Depression Society Guidelines for Bipolar Disorder [24].

### 2.3 Selection of Expert Panel Members

The expert panel consisted of the following three groups: patients living with bipolar disorder, family members and healthcare professionals with experience in treating bipolar disorder. The patient panel members included at least one person each who had been diagnosed with bipolar I or II disorder, who had not been recently hospitalised, taken a leave of absence or resigned due to worsening symptoms, and whose participation in this study was considered not to affect their symptoms by their physicians. We asked a patient advocacy group to introduce patients who met the above criteria. In addition, we asked a company particularly active in disseminating information about the experiences of patients with bipolar disorder, which was identified through an Internet search, to introduce eligible patients. The family panel members were also recruited through the patient advocacy group. Since symptoms of bipolar disorder may affect patient–family relationships [25], we selected family panel members who were unrelated to the recruited patients. As for healthcare professionals, since their expertise needed to cover not only drug treatment but also daily life consultation and support, we decided to recruit psychiatrists certified as designated mental health physicians, certified clinical psychologists and certified mental health social workers with experience in consultation, guidance and support for patients with bipolar disorder and their families. Healthcare professionals were recruited through the network of the Graduate School of Medicine, Kyoto University. We acknowledged that patients, family members and healthcare professionals are not exclusive categories. We requested the expert panel members to contribute from their assigned perspective. We aimed to recruit up to three experts per group, totalling up to 15 panel members. While it may raise concerns about the generalisability and robustness of the consensus, the appropriate number of participants for face‐to‐face meetings is 7–15 [26], ensuring that each person's opinion can be expressed.

### 2.4 First Individual Rating

Since information necessary for patients living with bipolar disorder varies depending on their stage of recovery and environment, we focused on the initiation phase of maintenance treatment as the timing when the essential information set will be utilised. Table 1 shows a vignette created before the first individual rating for panel members to understand the disease scenario. Based on the similarity of the number of items to be rated and the objective of prioritising the knowledge needed, we adopted the scaling of a previous study that determined the prioritisation of content in medical education [27]. Panel members rated each item of essential information on a 4‐point scale (definitely must = 3 points, probably should = 2 points, possibly could = 1 point or not necessary = 0 point). Table 2 shows the rating criteria. The score for each rating was multiplied by the number of panel members selecting the rating to obtain the total score. Panel members were briefed in advance before the rating on the timing of the disease and the four‐point scale so that they would have a common understanding of the rating criteria.　A consensus threshold was set at ≥ 51% of panel members rating an item as ‘definitely must’. This threshold was chosen based on the recommendation of a complementary use of three measures to assess consensus [28].

**Table 1:** Vignette: Description of the timing at which the developed information set is expected to be utilised.

Patient A has been seeing a doctor for depression on a regular basis, but was in distress as it did not seem to be getting any better. When A visited another hospital as recommended by a family member and explained how the conditions have been, the doctor said it was highly likely that A had bipolar disorder. A had never heard of bipolar disorder but noticed that some of the symptoms the doctor listed as specific examples of manic symptoms were what A had been experiencing. After changing the treatment, A got out of depression and is now able to perform everyday activities. A is hoping to return to work. A, who understands the diagnosis of bipolar disorder, was told by the doctor that long‐term treatment would be necessary. However, with the symptoms resolved, A is now wondering if it is necessary to continue the treatment.

**Table 2:** Rating and scoring.

Rating	Explanation	Score
Definitely must	Information that the assumed user must know. If the user does not know this, it will be very difficult/impossible to ‘have a social life while controlling the symptoms’	3
Probably should	Very useful information for the assumed user to know to actively/easily ‘have a social life while controlling the symptoms’	2
Possibly could	Information that is useful for the assumed user to ‘have a social life while controlling the symptoms’ but does not have as big an impact as ‘definitely must’ or ‘probably should’ information	1
Not necessary	Information that does not directly impact the assumed user to ‘have a social life while controlling the symptoms’	0

### 2.5 First Panel Meeting

The first panel meeting was held separately for patients, family members and healthcare professionals. For each item, the total score and the number and percentage of panel members by rating scale were presented in a table. The panel members reviewed the list of items presented in descending order of total score, exchanged opinions and selected the ‘definitely must’ items. Revisions to terminology and addition and consolidation of items suggested by the panel members in the first evaluation were discussed and reflected in the items.

### 2.6 Second Individual Rating

Panel members evaluated the items a second time. The list of candidate items was reviewed and streamlined based on feedback from the first panel meeting. All items were presented in the order of highest to lowest total score, and panel members rated each item on the 4‐point scale as described above with the same consensus threshold.

### 2.7 Second Panel Meeting

The list of items was presented in descending order of total score, along with the results from the first panel meeting for reference. Panel members reviewed the items presented, exchanged opinions and selected the ‘definitely must’ items.

### 2.8 Finalisation of the Essential Information Set

Items selected as ‘definitely must’ in the first and second panel meetings were included in the essential information set. The inclusion of items rated ‘definitely must’ in either evaluation was discussed and decided in the second panel meeting. The panel also considered including items not rated as ‘definitely must’ in the two ratings before finalising the essential information set.

### 2.9 Statistical Analysis

A descriptive statistical analysis was performed at each individual rating. We calculated percentages by each item using Microsoft Excel.

## 3 Results

### 3.1 Search Results

We identified as sources of information for patients and their families the websites of two Japanese academic societies (Japanese Society of Mood Disorders [29] and Japanese Society of Psychiatry and Neurology [30]), one national government agency (Ministry of Health, Labour and Welfare [31]), and three Japanese pharmaceutical companies that market drugs for bipolar disorder [32, 33, 34]. We also found three English language sources, in which information was provided by non‐Japanese academic societies or governments (International Society of Bipolar Disorders [35], the Royal Australian & New Zealand College of Psychiatrists [36] and the National Health Service UK [37]). In addition to the above, we referred to the content of a psychoeducation programme [24] recommended in the 2020 Japanese Depression Society Guidelines for Bipolar Disorder [25].

**Figure 2:**
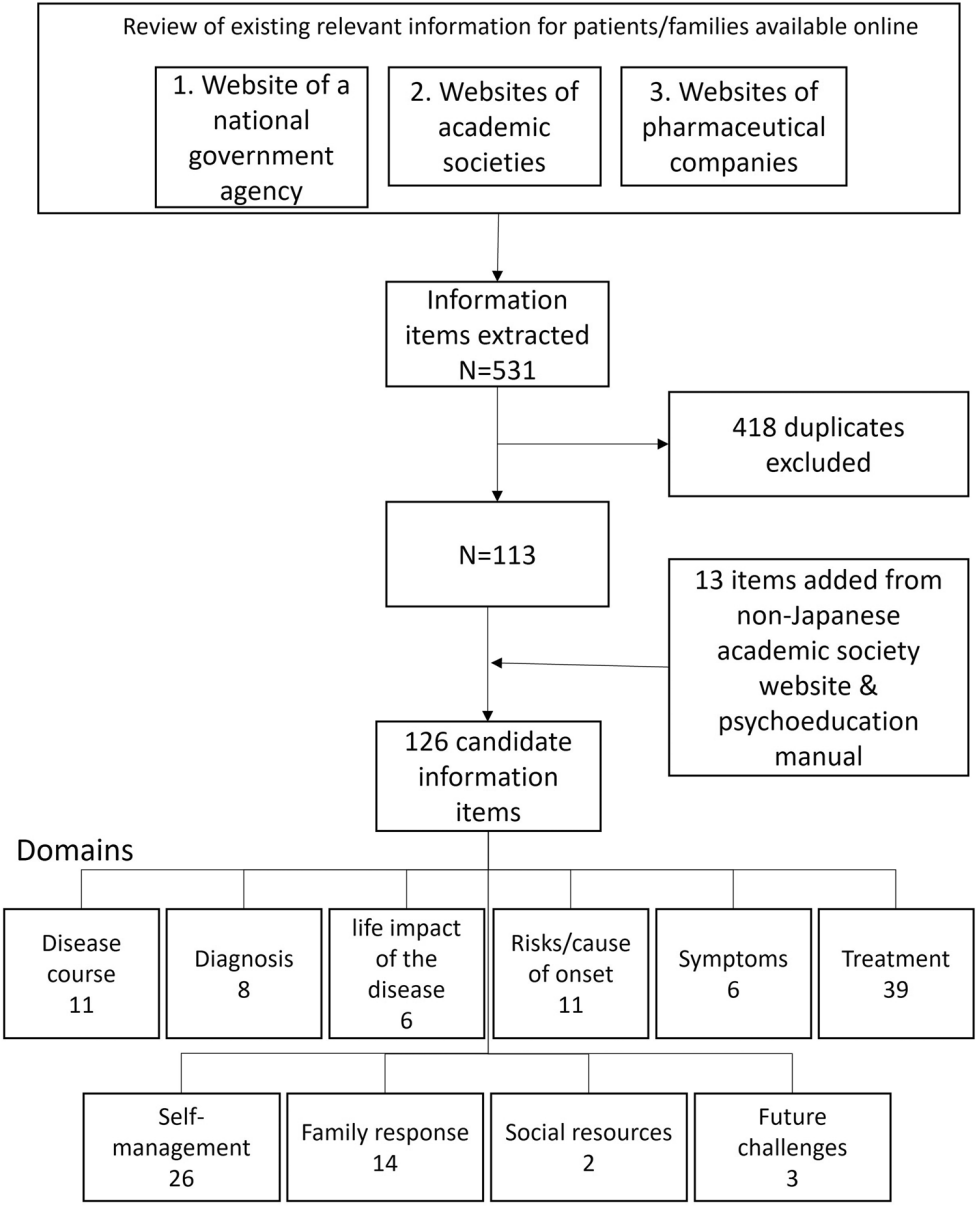
Generation of an initial candidate set of essential information.

To develop a list of candidate items, information on websites of the two domestic psychiatry societies, the Ministry of Health, Labour and Welfare, and three pharmaceutical companies was divided into sentence units. Next, duplicate contents were removed from the items listed, and similar contents were combined. Three psychiatrists (T.A., Y.S. and K.I.) and T.N. reviewed the items. A total of 126 items were identified as candidates for the essential information set and were classified according to the six domains of the BDKS (risk/cause of onset, diagnosis, disease course, symptoms, treatment and the life impact of the disease). There were 45 items that did not fit into any of the six domains, and four new domains (self‐management, family response, social resources and future challenges) were created (Figure 2).

### 3.2 Panel Members

A total of 11 expert panel members were selected, including three patients (one with type I bipolar disorder and two with type II bipolar disorder), three family members (two were currently living with a patient and one had previously lived with a patient), three psychiatrists certified as designated mental health physicians, one certified clinical psychologist, and one certified mental health social worker. The patients and family members were unrelated. Each panel member signed a consent form to participate in the present study.

### 3.3 First Individual Rating and Panel Meeting

The individual rating was conducted between 17 December 2022 and 31 January 2023. Responses were obtained from all 11 panel members, and 126 items were rated. Out of the total score of 33, the highest score was 31, the lowest was 10 and the average was 21. Twenty‐five items were rated as ‘definitely must’ by ≥ 51% of panel members. There were no items that were rated as ‘not necessary’ by more than 50% of the panel members.

The first round of panel meetings was conducted online in three separate groups (i.e., patients, family members and healthcare professionals) between 15 February and 2 March 2023, for 3 h each. One patient panel member was unable to attend the meeting and was interviewed at a later date. Items were presented in the order of highest to lowest total score, and each group decided which items should be selected as ‘definitely must’. A total of 34 items were selected as ‘definitely must’, including 21 of the 25 items rated as ‘definitely must’ in the first individual rating (four items were not selected as ‘definitely must’ by any of the three groups). 13 items that were not rated as ‘definitely must’ by ≥ 51% of panel members in the first individual rating were considered ‘definitely must’ by one of the three groups in the panel meeting.

During the panel meeting, suggestions were made to modify the wording of some items, including the following: the phrase ‘a threat to increase the possibility of relapse if treatment is not continued’; an ambiguous description about controlling symptoms; a categorical statement that acceptance of the disease is a prerequisite for continued treatment; and a statement that the family is obligated to take care of the patient. Accordingly, revisions were made based on the suggestions, and duplicate contents were also reviewed, leading to the integration of 30 items into other similar items, bringing the total number of items from 126 to 96. The 34 items selected as ‘definitely must’ were combined into 26 items.

### 3.4 Second Individual Rating

The second individual rating was conducted between 4 and 22 June 2023, and responses were obtained from all 11 panel members. A total of 96 items were rated, with the highest, lowest and average scores being 32, 5 and 16, respectively. Of the 26 items selected as ‘definitely must’ in the first panel meeting, 17 were rated as ‘definitely must’ by ≥ 51% of panel members in the second individual rating. There were three items that were rated as ‘not necessary’ by more than 50% of the participants (Supplement 1).

### 3.5 Second Panel Meeting and Finalisation of the Essential Information Set

The second panel meeting was a 2‐h online meeting held jointly among patients, family members and healthcare professionals. Three members (two family members and one healthcare professional) were absent but were provided with a separate opportunity to share their opinions at a later date.

**Figure 3:**
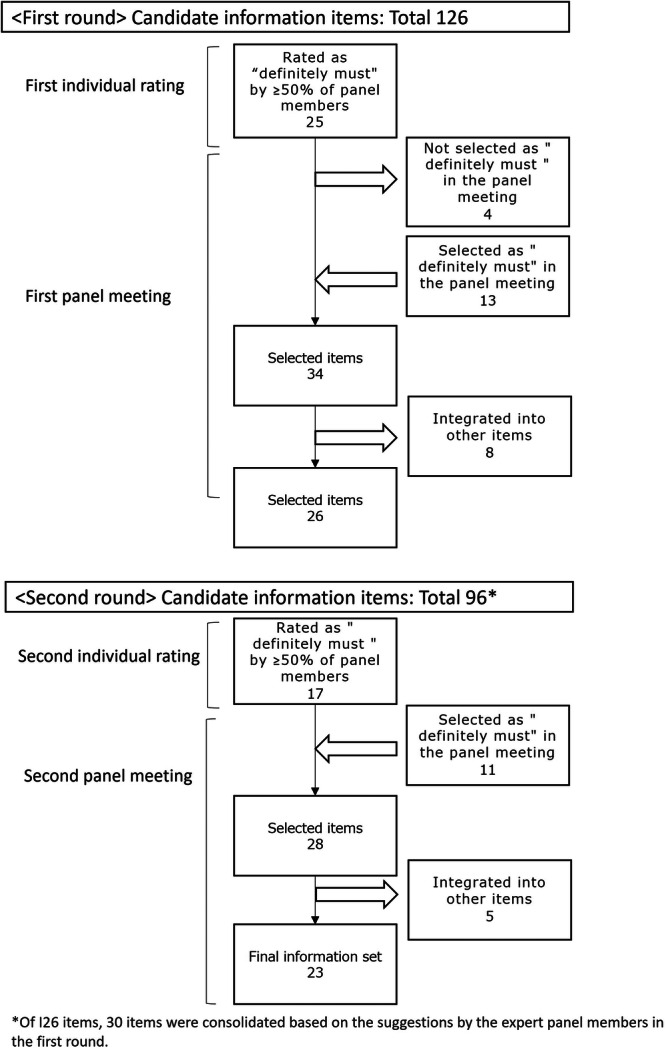
Flow of the individual ratings and panel meetings.

First, 17 items rated as ‘definitely must’ in both the first panel meeting and the second individual rating were included in the essential information set. Next, the panel reviewed items that were selected as ‘definitely must’ in the first panel meeting but were not rated as ‘definitely must’ in the second individual rating by ≥ 51% of panel members; among those, four items were ultimately included in the set. Finally, the panel reviewed items other than those selected in the order of highest to lowest score and discussed whether any should be included; as a result, seven items were added to the set. Of the 28 items, five were integrated into other similar items due to overlapping contents, and 23 items were selected to be included in the final essential information set. Figure 3 shows the flow of the individual ratings and panel meetings. The developed essential information set and the scores and ratings of each item are presented in Tables 3 and 4. Since most of the information for patients and their families has been created primarily by healthcare professionals, we aggregated ratings of patients and family members to understand the differences between healthcare professionals and non‐professionals. The original scores are presented in a separate table (Supplement 2). To enhance practical usability, we reorganised the essential information set by domain headings (Supplement 3).

**Table 3:** List of essential information set.

Number	Domain	Content
1	Self‐management	By keeping both symptoms of mania and depression at a manageable level to the extent possible and maintaining that condition for a long time, you can have a social life while coping well with bipolar. To achieve this, it is necessary to continue treatment. Dropping out of treatment is often a trigger for worsening symptoms.
2	Treatment	There may be times when you wish you could stop taking medications for various reasons, such as that the side effects are painful or they do not seem effective, or you may want to reduce doses because you have gotten better. However, some medications can make you feel sicker if you discontinue abruptly, so please tell your doctor what you want, rather than making decisions on your own. It is also a good idea to ask in advance when to reduce the amount of medication and what to do if you have doubts about your medication, such as side effects. If it is difficult to talk to your treating psychiatrist, you can also seek advice from pharmacists, nurses or mental health social workers.
3	Family response	We ask family members to please first take care of their own physical and mental health. There are things that family members can do, but do not think ‘I have to do it’ and never overdo it. Upon understanding this, you can learn what the signs of manic episodes are like while the patient's symptoms are stable, and decide specifically what actions to take within your capabilities when signs appear again. If you can implement this, it will be more likely that the worsening of manic symptoms can be avoided, and the social impact of manic symptoms can be reduced.
4	Symptoms	Symptoms of mania include the following: – Overly elated mood, or a state of excitement or being irritable – Having almost no sleep does not bother the patient – Improved opinion of oneself – Become more talkative than usual – All kinds of thoughts come to mind one after another – Lose track of things easily – Become active, and when severe, become hyperactive – Become enthusiastic about fun things (shopping extravagantly, being sexually uninhibited, investing in unrealistic business, etc.) even though it is obvious that this would cause trouble later These symptoms overlap and persist for more than a few days. The patient feels great, but people around the patient notice how clearly different he/she is compared to normal. Because of these symptoms, trouble occurs at work, in relationships or financially. It is a characteristic of manic symptoms that, if anything, people around the patient are more affected than the patient him/herself. The symptoms are not easily distinguishable from normal mood ups and downs, and depending on the duration of the symptoms and the degree of disruption to daily life, the doctor will comprehensively determine whether it is bipolar disorder and whether treatment is necessary.
5	Family response	To support the patient, family members and people around the patient can try the following things to the best of their ability: – Support when the patient is experiencing difficulty with everyday activities – Support the patient to continue treatment and manage medication – Accompany clinic visits, record and tell the primary physician the progress of symptoms, how the patient is at home, and so forth. – Support the patient to contact school, workplace and so forth to prepare for returning.
6	Course	There are various reasons why symptoms worsen, which may differ from person to person. These include ‘forgetting to take medicine’, ‘disturbance of sleep and wakefulness’, ‘setting goals too high’, ‘stress arising from relationships with other people’, ‘seasonal changes’ (e.g., depressed in winter and manic in summer), and for women, ‘childbirth’ (e.g., waves of emotion after childbirth) and ‘menstrual cycle’ (e.g., proneness to depression before menstruation). Some signs of worsening symptoms may be noticed only by the patient, while others may be more apparent to people around them.
7	Symptoms	A mixed episode involves the presence of both depressive and manic symptoms at the same time. This state is associated with a particularly high risk of suicidal ideation and behaviour, as individuals may feel hopeless while also having increased energy or impulsivity. If you notice signs of a mixed episode, please consult your doctor, as treatment adjustments may be necessary.
8	Social resources	In addition to the symptoms of bipolar, there may be other concerns in everyday living. Consultation services are available, including those offered by the primary psychiatrist, the community health centre and the mental health welfare centre. They will be able to refer you to appropriate professionals or welfare services depending on the nature of concern, whether it is about financial matters, social connection, life support or returning to work.
9	Treatment	In addition to continuing your medication, you can do all kinds of things in your life to keep the wave of symptoms calm. Specifically, these include: – Keeping regular hours – Giving yourself enough sleep time – Adopting healthy eating habits – Exercising moderately, and – Preventing stress from accumulating too much
10	Life impact of the disease	Depressive symptoms are painful for the patient, whereas manic and hypomanic symptoms often feel as if one is in good shape. On the other hand, family members and people around the patient often feel burdened by the manic symptoms of the patient. There may be discrepancies in how manic symptoms and depressive symptoms are perceived by the patient versus people around the patient, including family members.
11	Self‐management	When manic symptoms and depressive symptoms become pronounced, you will not be able to see your condition objectively. While your symptoms are stable, you should talk to your family and people around you to discuss what symptoms are expected, and when they appear, what responses you would want them to take, as this will help keep the waves of symptoms calm. Although it is helpful to look back and write down what triggered the worsening of your symptoms in the past, so that you can learn what the signs are, this may also upset you. Please consult your doctor to decide on the appropriate method and timing to reflect.
12	Family response	If you are worried that your symptoms may suddenly worsen, it will give you peace of mind to check with your doctor in advance, such as how to contact him/her outside of clinic hours. Some prefectures have a reception desk set up for emergency psychiatric consultation.
13	Treatment	Depressive symptoms are very painful, so the patient will feel that treatment is needed; on the other hand, when the patient has hypomanic symptoms, neither the patient nor family members may find them so burdensome and think treatment is not necessary. However, a manic episode is often followed by a depressive episode. Appropriate treatment of even mild manic symptoms can prevent or reduce subsequent depression symptoms.
14	Family response	When depressive symptoms worsen, the patient may start to have suicidal ideation. This is also one of the symptoms of bipolar. As a family member, you may not know how to respond to this, but do not carry the burden all by yourself and consult the treating psychiatrist.
15	Symptoms	Depressive symptoms include the following: 1. You feel depressed all day, and your spirit sinks 2. You lose interest in most things and cannot enjoy what you would normally have fun doing 3. Your appetite decreases (or increases), and you lose weight (or gain weight) 4. You have sleep problems, such as insomnia, which includes difficulty falling asleep, waking up in the middle of the night, waking up early in the morning or sleeping too much 5. The way you speak or move becomes dull, or you become irritable and restless 6. You feel tired easily and have low energy 7. You feel ‘I am worthless’ and blame yourself 8. You find it difficult to concentrate on things or to make a decision 9. You have thoughts like ‘I want to disappear from this world’ and ‘I want to die’. These symptoms overlap and persist all day, every day, for weeks.
		These are very painful symptoms for the patient, but others sometimes misunderstand and think that the patient is just being lazy. In particular, in the case of bipolar disorder, the patient finds the gap between depressive and manic symptoms painful.
16	Treatment	For depression, the goal of treatment is to improve the symptoms, but for bipolar disorder, treatment is aimed at keeping the waves of symptoms as calm as possible. There are central drugs (mood stabilisers) for that purpose. In addition, different drugs are available that are effective for each condition, such as when you need to improve manic symptoms, when you need to improve depressive symptoms, and when you can't sleep, and several drugs are used in combination depending on the symptoms and conditions. It will make it easier to consult with your doctor to explain which drugs are central to keep the waves of symptoms calm, and for what purpose other drugs are prescribed.
17	Family response	If manic symptoms are severe, they may place an increasing burden on the family and people around, such as handling social troubles. Therefore, you may need to be aware that they may experience difficult emotions towards you that they may harbour negative feelings against you.
18	Risk/cause	Bipolar disorder is a brain disorder characterised by recurrent manic episodes and depressive episodes.
19	Treatment	Even if you have bipolar disorder, you can get pregnant and give birth under the guidance of professional psychiatrists and by adjusting your medication. It is also helpful to consult your doctor or ask other patients about their experience of giving birth in a patients' association (PA) meeting, and so forth.
20	Diagnosis	If you suffer from repeated depressive episodes which do not seem to be getting any better, it is possible that you have bipolar disorder. If you look back carefully with your family or people around you on courses leading to the appearance of depressive symptoms, you may find that you have had manic symptoms; this can sometimes lead to a diagnosis of bipolar disorder.
21	Life impact of the disease	It is difficult to accept the diagnosis and prepare yourself for treatment. It is natural to experience complicated feelings before you are able to accept bipolar.
22	Self‐management	Sometimes other patients may understand your feelings about bipolar, family and so forth, as they themselves have the disease. It is also a good idea to participate in PA meetings.
23	Family response	A patient with depression has low energy and needs rest. Also, as the patient has lost the feeling of being happy, family members should refrain from encouraging or inviting the patient out for a distraction. Moreover, it is a burden for the patient to be asked if he/she is okay over and over. It may help if family members try to maintain some emotional flexibility and avoid becoming overly anxious.

**Table 4:** Ratings and scores of the final essential information set.

Number	Total score (2nd meeting)	Rating (2nd meeting)	Number (%) of members selecting ‘definitely must’/Healthcare professionals (*n* = 5) (2nd meeting)	Number (%) of members selecting ‘definitely must’/Patients and family members (*n* = 6) (2nd meeting)	Number (%) of members selecting ‘definitely must ’/Overall (*n* = 11) (2nd meeting)	Total score (1st meeting)	Rating (1st meeting)	Number (%) of members selecting ‘definitely must’/Healthcare professionals (*n* = 5) (1st meeting)	Number (%) of members selecting ‘definitely must ’/Patients and family members (*n* = 6) (1st meeting)	Number (%) of members selecting ‘definitely must’/Overall (*n* = 11) (1st meeting)	Total score (1st + 2nd meetings)
1	32	Definitely must	5 (100%)	5 (83%)	10 (91%)	31	Definitely must	4 (80％)	6 (100%)	10 (91%)	63
2	31	Definitely must	4 (80%)	5 (83%)	9 (82%)	30	Definitely must	4 (80％)	5 (83%)	9 (82%)	61
3	31	Definitely must	4 (80%)	5 (83%)	9 (82%)	30	Definitely must	5 (100%)	3 (50%)	8 (73%)	61
4	30	Definitely must	5 (100%)	4 (67%)	9 (82%)	31	Definitely must	5 (100%)	4 (67%)	9 (82%)	61
5	30	Definitely must	4 (80%)	4 (67%)	8 (73%)	29	Definitely must	4 (80％)	4 (67%)	8 (73%)	59
6	30	Definitely must	4 (80%)	4 (67%)	8 (73%)	29	Definitely must	4 (80％)	3 (50%)	7 (64%)	59
7	31	Definitely must	4 (80%)	5 (83%)	9 (82%)	27	Definitely must	2 (40%)	4 (67%)	6 (55%)	58
8	28	Definitely must	4 (80%)	4 (67%)	8 (73%)	28	Definitely must	3 (60%)	3 (50%)	6 (55%)	56
9	29	Definitely must	2 (40%)	6 (100%)	8 (73%)	26	Definitely must	2 (40%)	3 (50%)	6 (55%)	55
10	28	Definitely must	3 (60%)	5 (83%)	8 (73%)	27	Definitely must	4 (80％)	2 (33%)	6 (55%)	55
11	28	Definitely must	2 (40%)	4 (67%)	6 (55%)	27	Definitely must	2 (40%)	4 (67%)	6 (55%)	55
12	27	Definitely must	3 (60%)	4 (67%)	7 (64%)	27	Definitely must	3 (60%)	4 (67%)	7 (64%)	54
13	27	Definitely must	1 (20%)	5 (83%)	6 (55%)	26	Definitely must	3 (60%)	3 (50%)	6 (55%)	53
14	26	Definitely must	1 (20%)	5 (83%)	6 (55%)	25	Definitely must	2 (40%)	2 (33%)	4 (36%)	51
15	24	Definitely must	1 (20%)	2 (33%)	3 (27%)	25	Definitely must	3 (60%)	1 (17%)	4 (36%)	49
16	24	Definitely must	0 (0%)	3 (50%)	3 (27%)	25	Probably should	2 (40%)	2 (33%)	4 (36%)	49
17	21	Definitely must	1 (20%)	2 (33%)	3 (27%)	24	Probably should	1 (20%)	3 (50%)	4 (36%)	45
18	20	Definitely must	0 (0%)	2 (33%)	2 (18%)	25	Probably should	1 (20%)	4 (67%)	5 (45%)	45
19	22	Definitely must	0 (0%)	3 (50%)	3 (27%)	23	Probably should	1 (20%)	2 (33%)	3 (27%)	45
20	20	Definitely must	0 (0%)	2 (33%)	2 (18%)	24	Definitely must	2 (40%)	3 (50%)	5 (45%)	44
21	18	Definitely must	1 (20%)	3 (50%)	4 (36%)	25	Definitely must	3 (60%)	3 (50%)	6 (55%)	43
22	19	Definitely must	0 (0%)	1 (17%)	1 (9%)	22	Probably should	1 (20%)	2 (33%)	3 (27%)	41
23	19	Definitely must	0 (0%)	2 (33%)	2 (18%)	20	Probably should	0 (0%)	2 (33%)	2 (18%)	39

## 4 Discussion

In this study, a 23‐item essential information set, consisting of nine domains for patients and their families to live well with bipolar disorder, was created through consensus building to incorporate the perspectives of all parties involved, that is, patients, family members and healthcare professionals, regarding which information should be prioritised.　The selected 23 items were concordant with ‘the minimum essential information’ suggested by the Japanese clinical practice guideline [7].

From healthcare professionals' view, the BDKS comprises the six domains as ‘symptoms’, ‘diagnosis’, ‘risk/cause of onset’, ‘disease course’, ‘treatment’ and ‘the life impact of the disease’ [18]. For the essential information set, we considered domains other than the above to be necessary for patients to live well with the disease. Specifically, we added ‘family response’, ‘self‐management’ and ‘social resources’ for the final essential information set. In BDKS, the treatment‐related items were the most numerous (7 of 25 items). Next were five items related to the life impact of the disease, followed by four items each related to diagnosis and disease course [18]. In the essential information set comprising 23 items, the order of numbers was as follows: family response‐related items, 6; treatment, 5; and self‐management and symptoms, 3 for each. Treatment‐related items were consistently deemed important. However, there was a noticeable difference between items prioritised by healthcare professionals and those emphasised through the participation of patients and family members. Their contributions, based on lived experience, highlighted aspects of daily life that may not be fully captured by clinical perspectives.

Among items in the essential information set, ‘lifestyle changes that can be made to stabilize symptoms’ (Table 3, No. 9), ‘reasons why it is better to treat manic symptoms even mild’ (No. 13) and ‘how to deal with patients when they express their desire to disappear’ (No. 14) were rated as ‘definitely must’ by 40%, 20% and 20% of healthcare professionals, respectively, and by 100%, 83% and 83% of patients/families, respectively (Table 4). These findings suggest that the emphasis placed on these items in terms of ‘living well with the disease’ differs between healthcare professionals and patients/families. This discrepancy might arise because patients and their families were primarily focused on their daily activities and strategies for managing the challenges they encountered in daily life.

During the consensus‐building process, patients and families pointed out that it is difficult to understand the terms ‘relapse’ and ‘control’, which are commonly used by healthcare professionals in the context of treatment for bipolar disorder. In addition, patients and families indicated the need to consider changing statements that urge patients/families to take action (e.g., ‘Stopping treatment increases the probability of relapse’), which are often included in information provided to people with bipolar disorder. As for the expression ‘acceptance of the disease is a prerequisite for continued treatment’ has elicited both opinions from patients and families: ‘accepting it is really difficult, so making it the first step sets the bar too high’ and ‘facing this illness is important, so this expression is appropriate’. Thus, when disseminating information, the wording must be carefully chosen to reflect the viewpoints of the service users, especially the patients and their families.

This study does not meet the definition of patient and public involvement (PPI), which is commonly understood as research being carried out ‘with’ or ‘by’ members of the public rather than ‘to’, ‘about’ or ‘for’ them [38]. Patients and family members were not involved in designing or shaping the study, but rather participated as panel members in the Delphi process. At the same time, the research question was informed by prior informal conversations with patient advocacy groups and peer‐led meetings. While these interactions did not constitute formal involvement, they helped the research team to better understand the lived experiences and needs of patients and families.

The 23‐item essential information set developed in this study provides the minimum necessary information for all parties concerned, including patients, family members and healthcare professionals. While this set is designed to be comprehensive, the application of specific items may vary depending on the audience. For instance, advice to patients may focus more on self‐management and treatment adherence, while advice to family members may emphasise support strategies and understanding symptoms. During our face‐to‐face discussion, we talked about whether it would be better to separate information intended for families. The conclusion was that even information for families should be known by the patients, and it would be better not to give the impression that there is information being hidden from the patients by separating it.

It is also important to note that while this set represents the minimum, it may not be sufficient for everyone. Additional information needed by patients and their families varies based on factors such as background, environment and literacy. In the first round, there were no items rated as ‘not necessary’ by more than 50% of the participants. In the second round, three items were rated as such. This suggests that items outside of this essential information set are also important or needed depending on patients, and the prioritisation of information to be provided should be individualised. It is also crucial to consider the timing, amount and setting in which healthcare professionals deliver information and assess understanding on a case‐by‐case basis. Rather than testing knowledge, this set of items should be used to identify the information or support that each patient requires, aligning the understanding of patients, families and healthcare professionals to cope with the illness together. At the same time, the study highlights the gap in psychoeducational resources in routine clinical practice, a challenge that is not unique to Japan. By providing a structured and prioritised information set, our work offers a practical solution that can be utilised by healthcare professionals globally to enhance patient care. It should be noted that the expression and wording of the information should be customised based on the language and cultural context of each country.

This study has some limitations. First is the characteristics of the panel. Although the composition of the present panel was designed to ensure diversity in perspectives among healthcare providers, patients and family members, the absence of multiple panelists for each group may have limited the range of opinions within each group. While the panel size was determined to balance the need for in‐depth discussions with practical constraints, we acknowledge that both the limited number of participants and the selection of professional roles may constrain the generalisability of the findings.

Second, the sources of information are limited to information from government agencies, academic societies and pharmaceutical companies with medical supervision, which can be accessed via the Internet. This selection was based on the hypothesis that patient‐facing materials from trusted institutions broadly cover the essential knowledge needed to live with bipolar disorder. To ensure the comprehensiveness of the information set, we also referred to the psychoeducation manual, as it is specifically designed to support patient education. Other clinical resources, including internationally recognised guidelines and clinical manuals for psychotherapies, were not included because they did not meet these criteria. That said, some of the patient‐facing materials we reviewed did reference psychotherapies such as CBT and IPSRT, and related items were considered during the Delphi process, though they were not selected in the final set. Pharmaceutical company materials were included because they met these criteria and are commonly used in Japan to provide general information about disease states with medical supervision via their website. However, we acknowledge that the inclusion of such materials may raise concerns about potential bias. Although experiences and information shared by patients and their families are also available in literature and online platforms beyond the sources included in this review, the individuals we recruited were actively disseminating their own experiences as experts. Accordingly, it appears that the essential information was adequately captured. Third, as this study aimed to develop an essential information set through consensus building, the resulting content is not intended to serve as a universally sufficient resource for continuing treatment or preventing symptom recurrence. Its contribution to these outcomes requires further validation. Future research should explore how the information set can be implemented and communicated across diverse clinical and community settings, including its effects on treatment adherence, patient outcomes and satisfaction among patients, families and healthcare professionals. Finally, this information set reflected the current body of available evidence and the consensus of a multidisciplinary panel. Although the source of information we reviewed included distinctions between Bipolar I and II, this was not explicitly reflected in the final information set. This decision was based on feedback from patients and families, who noted that such distinctions were not always made clear during diagnosis or initial treatment discussions. While this may limit specificity, it reflects current clinical communication practices. It will be revised as new clinical evidence emerges, practice guidelines are updated, and the social systems and circumstances surrounding people living with bipolar disorder evolve.

## 5 Conclusion

We created a 23‐item essential information set across nine domains through consensus building among patients, family members and healthcare professionals regarding which information is necessary for living well with bipolar disorder. The 23 items could be shared among all parties involved at the initiation of the maintenance treatment phase and serve as the common basis for psychoeducation for bipolar disorder. Further studies should explore the practical effectiveness of the information set in diverse clinical settings.

## Author Contributions

Conception and design of the study: Rieko Nagata, Yoshitaka Nishikawa, Mayumi Toyama, Hiroshi Okada, Yoshimitsu Takahashi, Yu Sakagami, Eiji Suzuki, Norio Ozaki and Takeo Nakayama. Acquisition and analysis of data: Rieko Nagata, Takashi Amagasa, Takashi Okura, Kayoko Ichikawa, Yu Sakagami and Takeo Nakayama. Drafting the manuscript or figures: Rieko Nagata, Yu Sakagami and Takeo Nakayama. Manuscript writing: All authors. Final approval of manuscript: All authors. Accountable for all aspects of the work: All authors.

## Use of Generative AI Tools

In preparing this manuscript, the authors used generative AI tools to assist with language editing and phrasing improvements. All content generated by these tools was reviewed and verified by the authors, who take full responsibility for the final manuscript.

## Ethics Statement

Ethical approval for the study was granted by the Kyoto University Graduate School of Medicine Faculty of Medicine Medical Ethics Committee (R3310‐1).

## Consent

All participants signed a consent before participation in the study.

## Conflicts of Interest

Rieko Nagata is a full‐time employee of Eli Lilly Japan K.K. and a minor shareholder of Eli Lilly and Company. This study was conducted independently from the company.

Takashi Okura has received research grants and travel support from Okayama Prefectural University and Japan Society for the Promotion of Science and is a chairperson of Soja City Council for Promotion of Policies for Persons with Disabilities and a committee member of Japan suicide countermeasures promotion centre and Okayama Psychiatric Medical Center.

Yoshitaka Nishikawa has received research grants from the Japan Medical Association and donations from Datack and CancerScan.

Hiroshi Okada has received research grants from I&H Co. Ltd. and KRAFT Inc. and donation from Neo Plus Pharma Co. Ltd. and YUYAMA Co. Ltd.

Eiji Suzuki has received a research grant from Mochida Pharmaceuticals Co. Ltd.

Norio Ozaki has received grants from Sumitomo Pharma, Eisai, Otsuka, Shionogi, Mochida, KAITEKI, Takeda, Nihon Medi‐Physics, Eli Lilly Japan, Mitsubishi Tanabe and DAIICHI SANKYO; consulting fees from Sumitomo Pharma, Taisho Pharma, Boehringer Ingelheim, Otsuka and Mochida; and payments or honoraria for lectures, presentations, speakers bureaus, manuscript writing or educational events from Sumitomo Pharma, Eisai, Otsuka, Mochida, Takeda, Meiji Seika Pharma, EA Pharma, MSD, Lundbeck Japan, Viatris, Kyowa Kirin and TSUMURA.

Takeo Nakayama has received research grants from I&H Co. Ltd., Cocokarafine Co. Ltd., Konica Minolta Inc. and NTT DATA; consulting fees from Otsuka Pharmaceutical Co., Takeda Pharmaceutical Co., Johnson & Johnson K.K. and AstraZeneca plc.; honoraria from Pfizer Japan Inc., MSD K.K., Chugai Pharmaceutical Co., Takeda Pharmaceutical Co., Janssen Pharmaceutical K.K., Boehringer Ingelheim International GmbH, Eli Lilly Japan K.K., Maruho Co. Ltd., Mitsubishi Tanabe Pharma Co., Novartis Pharma K.K., Allergan Japan, Novo Nordisk Pharma Ltd., TOA EIYO Ltd., AbbVie inc., Ono Pharmaceutical Co. Ltd., GSK plc., Alexion Pharmaceuticals Inc., Canon Medical Systems Co., Kowa Co. Ltd., Araya, Merck Co., Amicus Therapeutics Inc. Amgen Inc. and CSL Behring K.K.; stock options from Bon Bon Inc.; and donations from CancerScan, JMDC Inc. and Yuyama. All other authors declare no support from any organisation for the submitted work; no financial relationships with any organisations that might have an interest in the submitted work in the previous 3 years; and no other relationships or activities that could appear to have influenced the submitted work.

## Supporting information

0250811_vF_Supplement

## Data Availability

The data that support the findings of this study are available from the corresponding author upon reasonable request.
