# Supplementary material for: Development of an Essential Information Set for Supporting Life With Bipolar Disorder: A Modified Delphi Study With Patients, Families and Healthcare Professionals
Source: Health Expect. 2025 Oct 22;28(5):e70470. doi: 10.1111/hex.70470 (PMC12541233; doi:10.1111/hex.70470)
Supplement: Supplementary file 1 — 0250811_vF_Supplement [file HEX-28-e70470-s001.docx]

Supplement 1

Items that were rated as “not necessary” by more than 50% of the participants (2^nd^ rating)

| Number  Table 3. Essential information set | Domain | Content |
| --- | --- | --- |
| 1 | Diagnosis | There are quite a few people who, despite having depression, have their symptoms improve and regain normal levels of energy, only to be mistakenly diagnosed with bipolar disorder due to being perceived as mildly manic. |
| 2 | Risk/cause | Some of the genetic predispositions that make a person more susceptible to bipolar disorder may manifest as personality traits that are also associated with a higher risk of developing the disorder (e.g., being sociable, kind, active, and easily engrossed in activities). |
| 3 | Risk/cause | It is often the case that differences in responses to environmental factors that cause diseases are determined by genetic traits. It has also been found that many diseases and the effects of medications, which may not appear to be hereditary at first glance, are actually influenced by genetic differences. |

Supplement 2

The individual ratings from each group (patient and family)

| Number  Table 3. Essential information set | Number (%) of members selecting "definitely must"/  patients  (n=3) (2nd meeting) | Number (%) of members selecting "definitely must"/ family members  (n=3) (2nd meeting) | Number (%) of members selecting "definitely must"/  patients  (n=3) (1st meeting) | Number (%) of members selecting "definitely must "/ family members  (n=3) (1st meeting) |
| --- | --- | --- | --- | --- |
| 1 | 3 (100%) | 2 (67%) | 3 (100%) | 3 (100%) |
| 2 | 3 (100%) | 2 (67%) | 3 (100%) | 2 (67%) |
| 3 | 2 (67%) | 3 (100%) | 2 (67%) | 1 (33%) |
| 4 | 3 (100%) | 1 (33%) | 3 (100%) | 1 (33%) |
| 5 | 1 (33%) | 3 (100%) | 2 (67%) | 2 (67%) |
| 6 | 2 (67%) | 2 (67%) | 2 (67%) | 1 (33%) |
| 7 | 2 (67%) | 3 (100%) | 2 (67%) | 2 (67%) |
| 8 | 1 (33%) | 3 (100%) | 2 (67%) | 1 (33%) |
| 9 | 3 (100%) | 3 (100%) | 2 (67%) | 1 (33%) |
| 10 | 2 (67%) | 3 (100%) | 1 (33%) | 1 (33%) |
| 11 | 2 (67%) | 2 (67%) | 2 (67%) | 2 (67%) |
| 12 | 1 (33%) | 3 (100%) | 2 (67%) | 2 (67%) |
| 13 | 2 (67%) | 3 (100%) | 2 (67%) | 1 (33%) |
| 14 | 2 (67%) | 3 (100%) | 2 (67%) | 1 (33%) |
| 15 | 1 (33%) | 1 (33%) | 1 (33%) | 0 (0%) |
| 16 | 2 (67%) | 1 (33%) | 1 (33%) | 1 (33%) |
| 17 | 1 (33%) | 1 (33%) | 2 (67%) | 1 (33%) |
| 18 | 2 (67%) | 0 (0%) | 3 (100%) | 1 (33%) |
| 19 | 1 (33%) | 2 (67%) | 2 (67%) | 1 (33%) |
| 20 | 0 (0%) | 2 (67%) | 2 (67%) | 1 (33%) |
| 21 | 2 (67%) | 1 (33%) | 2 (67%) | 1 (33%) |
| 22 | 0 (0%) | 1 (33%) | 2 (67%) | 0 (0%) |
| 23 | 1 (33%) | 1 (33%) | 2 (67%) | 0 (0%) |

Supplement 3. List of essential information set by domain

**Symptoms**

| 4 | Symptoms of mania include the following:  - Overly elated mood, or a state of excitement or being irritable  - Having almost no sleep does not bother the patient - Improved opinion of oneself  - Become more talkative than usual - All kinds of thoughts come to mind one after another  - Lose track of things easily - Become active, and when severe, become hyperactive  - Become enthusiastic about fun things (shopping extravagantly, being sexually uninhibited, investing in unrealistic business, etc.) even though it is obvious that this would cause trouble later These symptoms overlap and persist for more than a few days. The patient feels great, but people around the patient notice how clearly different he/she is compared to normal. Because of these symptoms, trouble occurs at work, in relationships, or financially. It is a characteristic of manic symptoms that, if anything, people around the patient are more affected than the patient him/herself. The symptoms are not easily distinguishable from normal mood ups and downs, and depending on the duration of the symptoms and the degree of disruption to daily life, the doctor will comprehensively determine whether it is bipolar disorder, and whether treatment is necessary. |
| --- | --- |
| 7 | A mixed episode involves the presence of both depressive and manic symptoms at the same time. This state is associated with a particularly high risk of suicidal ideation and behavior, as individuals may feel hopeless while also having increased energy or impulsivity. If you notice signs of a mixed episode, please consult your doctor, as treatment adjustments may be necessary. |
| 15 | Depressive symptoms include the following:  (1) You feel depressed all day, and your spirit sinks (2) You lose interest in most things and cannot enjoy what you would normally have fun doing (3) Your appetite decreases (or increases), and you lose weight (or gain weight) (4) You have sleep problems, such as insomnia which includes difficulty falling asleep, waking up in the middle of the night, and waking up early in the morning, or sleeping too much  (5) The way you speak or move becomes dull, or you become irritable and restless (6) You feel tired easily and have low energy (7) You feel "I am worthless" and blame yourself (8) You find it difficult to concentrate on things or to make a decision  (9) You have thoughts like "I want to disappear from this world" and "I want to die." These symptoms overlap and persist all day, every day, for weeks.  These are very painful symptoms for the patient, but others sometimes misunderstand and think that the patient is just being lazy. In particular, in the case of bipolar disorder, the patient finds the gap between depressive and manic symptoms painful. |

**Risk/Cause**

| 18 | Bipolar disorder is a brain disorder characterized by recurrent manic episodes and depressive episodes. |
| --- | --- |

**Diagnosis**

| 20 | If you suffer from repeated depressive episodes which do not seem to be getting any better, it is possible that you have bipolar disorder. If you look back carefully with your family or people around you on courses leading to the appearance of depressive symptoms, you may find that you have had manic symptoms; this can sometimes lead to a diagnosis of bipolar disorder. |
| --- | --- |

**Course**

| 6 | There are various reasons why symptoms worsen, which may differ from person to person.  These include "forgetting to take medicine," "disturbance of sleep and wakefulness," "setting goals too high," "stress arising from relationships with other people," "seasonal changes" (e.g., depressed in winter, manic in summer), and for women, "childbirth" (e.g., waves of emotion after childbirth) and "menstrual cycle" (e.g., proneness to depression before menstruation). Some signs of worsening symptoms may be noticed only by the patient, while others may be more apparent to people around them. |
| --- | --- |

**Life impact of the disease**

| 10 | Depressive symptoms are painful for the patient, whereas manic and hypomanic symptoms often feel as if one is in good shape. On the other hand, family members and people around the patient often feel burdened by the manic symptoms of the patient. There may be discrepancies in how manic symptoms and depressive symptoms are perceived by the patient vs. people around the patient including family members. |
| --- | --- |
| 21 | It is difficult to accept the diagnosis and prepare yourself for treatment. It is natural to experience complicated feelings before you are able to accept bipolar. |

**Treatment**

| 2 | There may be times when you wish you could stop taking medications for various reasons, such as that the side effects are painful or they do not seem effective, or you may want to reduce doses because you have gotten better. However, some medications can make you feel sicker if you discontinue abruptly, so please tell your doctor what you want, rather than making decisions on your own. It is also a good idea to ask in advance when to reduce the amount of medication and what to do if you have doubts about your medication, such as side effects If it is difficult to talk to your treating psychiatrist, you can also seek advice from pharmacists, nurses, or mental health social workers. |
| --- | --- |
| 9 | In addition to continuing your medication, you can do all kinds of things in your life in order to keep the wave of symptoms calm. Specifically, these include:  - Keeping regular hours - Giving yourself enough sleep time - Adopting healthy eating habits - Exercising moderately, and  - Preventing stress from accumulating too much |
| 13 | Depressive symptoms are very painful, so the patient will feel that treatment is needed; on the other hand, when the patient has hypomanic symptoms, neither the patient nor family members may find them so burdensome and think treatment is not necessary. However, a manic episode is often followed by a depressive episode. Appropriate treatment of even mild manic symptoms can prevent or reduce subsequent depression symptoms. |
| 16 | For depression, the goal of treatment is to improve the symptoms, but for bipolar disorder, treatment is aimed at keeping the waves of symptoms as calm as possible. There are central drugs (mood stabilizers) for that purpose. In addition, different drugs are available that are effective for each condition, such as when you need to improve manic symptoms, when you need to improve depressive symptoms, and when you can't sleep, and several drugs are used in combination depending on the symptoms and conditions. It will make it easier to consult with your doctor to explain which drugs are central to keep the waves of symptoms calm, and for what purpose other drugs are prescribed. |
| 19 | Even if you have bipolar disorder, you can get pregnant and give birth under the guidance of professional psychiatrists and by adjusting your medication. It is also helpful to consult your doctor or ask other patients about their experience of giving birth in a patients association (PA) meeting, etc. |

**Self management**

| 1 | By keeping both symptoms of mania and depression at a manageable level to the extent possible and maintaining that condition for a long time, you can have a social life while coping well with bipolar. To achieve this, it is necessary to continue treatment. Dropping out of treatment is often a trigger for worsening symptoms. |
| --- | --- |
| 11 | When manic symptoms and depressive symptoms become pronounced, you will not be able to see your condition objectively. While your symptoms are stable, you should talk to your family and people around you to discuss what symptoms are expected, and when they appear, what responses you would want them to take, as this will help keep the waves of symptoms calm. Although it is helpful to look back and write down what triggered the worsening of your symptoms in the past, so that you can learn what the signs are, this may also upset you. Please consult your doctor to decide on the appropriate method and timing to reflect. |
| 22 | Sometimes other patients may understand your feelings about bipolar, family, etc. as they themselves have the disease. It is also a good idea to participate in PA meetings. |

**Family response**

| 3 | We ask family members to please first take care of your own physical and mental health. There are things that family members can do, but do not think "I have to do it" and never overdo it. Upon understanding this, you can learn what the signs of manic episodes are like while the patient's symptoms are stable, and decide specifically what actions to take within your capabilities when signs appear again. If you can implement this, it will be more likely that worsening of manic symptoms can be avoided, and the social impact of manic symptoms can be reduced. |
| --- | --- |
| 5 | To support the patient, family members and people around the patient can try the following things to the best of their ability: - Support when the patient is experiencing difficulty with everyday activities - Support the patient to continue treatment and manage medication  - Accompany clinic visits, record and tell the primary physician the progress of symptoms, how the patient is at home, etc. - Support the patient to contact school, workplace, etc. to prepare for returning. |
| 12 | If you are worried that your symptoms may suddenly worsen, it will give you peace of mind to check with your doctor in advance, such as how to contact him/her outside of clinic hours. Some prefectures have a reception desk set up for emergency psychiatric consultation. |
| 14 | When depressive symptoms worsen, the patient may start to have suicidal ideation. This is also one of the symptoms of bipolar. As a family member, you may not know how to respond to this, but do not carry the burden all by yourself and consult the treating psychiatrist. |
| 17 | If manic symptoms are severe, they may place an increasing burden on the family and people around, such as handling social troubles. Therefore, you may need to be aware that they may experience difficult emotions toward you and that they may harbor negative feelings against you. |
| 23 | A patient with depression has low energy and needs rest. Also, as the patient has lost the feeling of being happy, family members should refrain from encouraging or inviting the patient out for a distraction. Moreover, it is a burden for the patient to be asked if he/she is okay over and over. It may help if family members try to maintain some emotional flexibility and avoid becoming overly anxious. |

**Social resources**

| 8 | In addition to the symptoms of bipolar, there may be other concerns in everyday living. Consultation services are available, including those offered by the primary physician, the community health center, and the mental health welfare center. They will be able to refer you to appropriate professionals or welfare services depending on the nature of concern, whether it is about financial matters, social connection, life support, or returning to work. |
| --- | --- |
